# The Association Between Logging Steps Using a Website, App, or Fitbit and Engaging With the 10,000 Steps Physical Activity Program: Observational Study

**DOI:** 10.2196/22151

**Published:** 2021-06-18

**Authors:** Anna T Rayward, Corneel Vandelanotte, Anetta Van Itallie, Mitch J Duncan

**Affiliations:** 1 School of Health Medical and Applied Sciences Central Queensland University Rockhampton Australia; 2 School of Education College of Human and Social Futures University of Newcastle Callaghan Australia; 3 School of Medicine & Public Health College of Health, Medicine and Wellbeing University of Newcastle Callaghan Australia

**Keywords:** physical activity intervention, activity trackers, engagement, Fitbit, pedometer, eHealth, mobile phone

## Abstract

**Background:**

Engagement is positively associated with the effectiveness of digital health interventions. It is unclear whether tracking devices that automatically synchronize data (eg, Fitbit) produce different engagement levels compared with manually entering data.

**Objective:**

This study examines how different step logging methods in the freely available 10,000 Steps physical activity program differ according to age and gender and are associated with program engagement.

**Methods:**

A subsample of users (n=22,142) of the free 10,000 Steps physical activity program were classified into one of the following user groups based on the step-logging method: Website Only (14,617/22,142, 66.01%), App Only (2100/22,142, 9.48%), Fitbit Only (1705/22,142, 7.7%), Web and App (2057/22,142, 9.29%), and Fitbit Combination (combination of web, app, and Fitbit; 1663/22,142, 7.51%). Generalized linear regression and binary logistic regression were used to examine differences between user groups’ engagement and participation parameters. The time to nonusage attrition was assessed using Cox proportional hazards regression.

**Results:**

App Only users were significantly younger and Fitbit user groups had higher proportions of women compared with other groups. The following outcomes were significant and relative to the Website Only group. The App Only group had fewer website sessions (odds ratio [OR] −6.9, 95% CI −7.6 to −6.2), whereas the Fitbit Only (OR 10.6, 95% CI 8.8-12.3), Web and App (OR 1.5, 95% CI 0.4-2.6), and Fitbit Combination (OR 8.0; 95% CI 6.2-9.7) groups had more sessions. The App Only (OR −0.7, 95% CI −0.9 to −0.4) and Fitbit Only (OR −0.5, 95% CI −0.7 to −0.2) groups spent fewer minutes on the website per session, whereas the Fitbit Combination group (OR 0.2, 95% CI 0.0-0.5) spent more minutes. All groups, except the Fitbit Combination group, viewed fewer website pages per session. The mean daily step count was lower for the App Only (OR −201.9, 95% CI −387.7 to −116.0) and Fitbit Only (OR −492.9, 95% CI −679.9 to −305.8) groups but higher for the Web and App group (OR 258.0, 95% CI 76.9-439.2). The Fitbit Only (OR 5.0, 95% CI 3.4-6.6), Web and App (OR 7.2, 95% CI 5.9-8.6), and Fitbit Combination (OR 15.6, 95% CI 13.7-17.5) groups logged a greater number of step entries. The App Only group was less likely (OR 0.65, 95% CI 0.46-0.94) and other groups were more likely to participate in Challenges. The mean time to nonusage attrition was 35 (SD 26) days and was lower than average in the Website Only and App Only groups and higher than average in the Web and App and Fitbit Combination groups.

**Conclusions:**

Using a Fitbit in combination with the 10,000 Steps app or website enhanced engagement with a real-world physical activity program. Integrating tracking devices that synchronize data automatically into real-world physical activity interventions is one strategy for improving engagement.

## Introduction

### Background

Meeting or exceeding recommended physical activity levels is key for the prevention and management of noncommunicable diseases [1,2]. However, large proportions of the population do not meet these recommendations [3,4]. In response, web- and app-based programs to promote physical activity among adults have been developed [5]. Few of these interventions have been evaluated in real-world settings [6]. Relative to randomized controlled trials whose participants are rigorously screened, have repeated contact with trial staff, and may include participants who are motivated to change behaviors, interventions conducted in less controlled and real-world settings may have significantly different levels of usage, engagement, nonusage attrition (users stop interacting entirely), and behavior change [6]. However, the greater accessibility of real-world programs makes them valuable avenues to reach larger populations at a relatively low cost [7]. Furthermore, the way users engage with web- and app-based programs in ecologically valid circumstances may potentially impact the effectiveness of these programs [6].

Web- and app-based programs that promote physical activity frequently include self-monitoring as an effective behavior change technique [8]. Although pedometers are commonly and successfully used in activity promotion efforts [9], the emergence of wrist-worn activity trackers such as Fitbit has provided a convenient, reliable, and accurate alternative to pedometers for tracking step counts. Worldwide sales of health and fitness trackers have more than tripled from 2014 (26 million units) to 2017 (87 million units) [10]. The use of technology to track health is widespread, with 33% of the worldwide population across all age groups using a mobile app or a fitness tracking device in 2016 [11]. Australia had the second highest wearable fitness device adoption rate in the world in 2016, with 14% of the population owning at least 1 device [12]. Therefore, it is logical to integrate advanced activity trackers that can automatically synchronize activity behavior into web- and app-based programs [13].

A randomized controlled trial, the *TaylorActive* intervention, delivered the same web-based computer-tailored physical activity intervention to a Fitbit group and a non-Fitbit group [14]. Compared with the non-Fitbit group (which self-reported frequency of, and time spent in, mild, moderate, and strenuous physical activity on the website or app), the group using Fitbit activity trackers significantly increased in total weekly physical activity (mean total physical activity increase of 163.2 min/week; 95% CI 52.0-274.0) and weekly moderate-to- vigorous physical activity (mean moderate-to-vigorous physical activity increase of 78.6 min/week; 95% CI 24.4-131.9) after 3 months [14]. However, it is unclear how the addition of a physical activity tracking device impacts the use of free real-world physical activity promotion programs. Previous research has questioned whether there is a difference in the effectiveness of active (eg, manual entry of step count into a database) versus passive (eg, automatic entry of step count into a database by an activity tracking device requiring no cognitive attention) self-monitoring of behaviors. However, there is a paucity of research on this subject [15]. The lack of effort required to log in to a website or app to manually enter recorded physical activity may limit both the cognitive effort and subsequent focus on improving or maintaining an activity goal. It may also preclude opportunities to engage with other program features found on websites and apps, such as goal setting and social support features, which may consolidate behavior change [16,17]. Alternatively, the reduced burden of having to manually log steps may remove perceived barriers, such as time constraints, and subsequently provide more time to engage with program features and result in longer engagement before nonusage attrition occurs. Furthermore, having multiple options on how to self-monitor physical activity could overwhelm some users, but it may increase interest and better engagement in other users through enhanced accessibility of their preferred self-monitoring method. In addition, fitness trackers are used more commonly by women and younger adults, which may influence engagement with web-based health interventions [10,11,18].

The 10,000 Steps program is a free, publicly available program that aims to promote physical activity through the use of pedometers, activity trackers, a website, and an app [19]. The website and app have been available for public use since 2004 and 2012, respectively. A previous study examining user engagement with 10,000 Steps program found that program engagement was higher and nonusage attrition was lower among those who used the app or a combination of app and website compared with those using only the website [18]. However, the effect on engagement since the capacity to automatically record and sync step counts with the 10,000 Steps website and app using a Fitbit activity tracking device was introduced in January 2017 has not been assessed.

### Objectives

This study aims to examine whether users’ logging methods (Website Only, App Only, Fitbit Only, Web and App, and Fitbit Combination) differ according to age and gender as well as how different methods of logging steps in the 10,000 Steps physical activity program are associated with engagement with the program.

## Methods

### Participants and Procedures

The 10,000 Steps program was designed as a free, publicly available, whole-of-community program to increase physical activity among adults. The program, which is based on a socioecological framework, began in Rockhampton, Australia, and utilizes multiple strategies to promote physical activity [20]. Further details on the program design and development are available elsewhere [21,22]. In general, the program encourages users to accumulate physical activity each day and monitor their daily physical activity levels (actively or passively) by recording their pedometer, activity tracker–counted steps and/or time spent in physical activity using a web-based step log. The web-based step log is available to users on both the 10,000 Steps website and a smartphone app. Activity and/or steps logged using the app are automatically synchronized with the user’s account on the website [18]. Steps recorded via a Fitbit activity tracking device were also synchronized with the app and website. Users can also access additional program features, including monthly Challenges for individuals (users may choose from a selection of virtual journeys with predefined monthly step goals and receive feedback in relation to progress), team Tournaments (created by 10,000 Steps coordinators from workplaces, community organizations, or groups of friends that involve team-based virtual walking Challenges based on a set amount of time or a predefined journey), and virtual *friends* (which allow users to track one another’s progress and motivate each other) [18].

**Figure 1:**
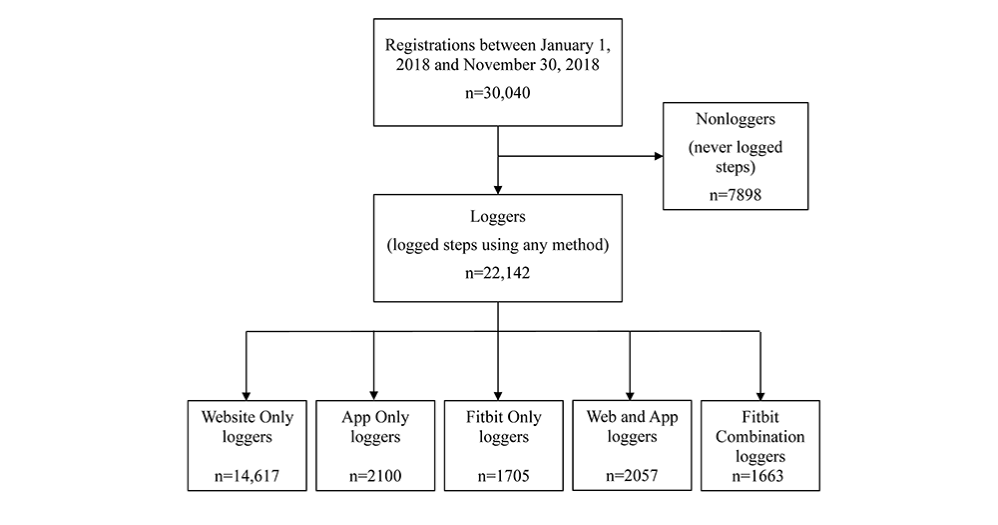
Classification of logging methods of new registrations to the 10,000 Steps program between January 1, 2018, and November 30, 2018.

In this study, participants were users of the 10,000 Steps program who registered between January 1, 2018, and November 30, 2018 (N=30,040). When registering with the 10,000 Steps program, users provided informed consent for the usage of their data for research purposes. Of these new registrations, 7898 never logged steps (referred to as *Nonloggers*) and were not included in the analyzed sample. The remaining 22,142 new users were classified into one of five user groups based on the method they used to log steps. Participants captured their daily step counts using either a Fitbit or some other device (eg, pedometers, phone apps, or non-Fitbit activity monitors). Then, they recorded their step counts on the 10,000 Steps platform by manually entering data via the website or app or via automatic syncing of their Fitbit with the platform. The methods used to log step entries were recorded on a website database. The user groups were as follows: Website Only (14,617/22,142, 66.01%; step count data entered *only via* the website), App Only (2100/22,142, 9.48%; step count data entered *only* via the app), Fitbit Only (1705/22,142, 7.7%; step count data entered *only* via the Fitbit), Web and App (2057/22,142, 9.29%; entered step count data using the website and the app), and Fitbit Combination (1663/22,142, 7.51%; step count data entered using a combination of Fitbit and website and/or app). The Fitbit Only group logged steps passively (although may have actively engaged with the website content otherwise) while the Fitbit Combination group logged steps passively and actively. All other groups actively logged the steps. Regardless of how users logged steps, they were all able to interact with the website content (Figure 1).

### Data Collection and Extraction

Data were extracted from the 10,000 Steps website database and Google Analytics to assess user characteristics, engagement, and platform usage. Data for new users were tracked from the time of registration (between January 1 and November 30, 2018) until December 31, 2018, which allowed a minimum of 31 days opportunity to observe all participants’ self-monitoring of physical activity.

### Measures

#### User Characteristics

The date of birth, gender, and registration date were recorded and stored in the website database. The date of birth was used to determine the age at the time of registration. Gender was categorized as male, female, or other.

#### User Engagement With Website and Program

User engagement data were extracted for the entire study period from January 1 to December 31, 2018. User groups were mutually exclusive and were defined based on the method of logging steps. However, irrespective of a user’s logging method, all users had the potential to access the website (eg, Fitbit Only users only logged steps via a Fitbit and had the opportunity to view the 10,000 Steps website and/or app). The website includes a variety of information, including library articles (eg, benefits of physical activity and strategies for increasing activity) with information on workplace and individual Challenges [22]. Users’ engagement with the 10,000 Steps website was assessed based on all pages or sections of the website by using the average number of sessions (using the website to interact with content; eg, viewing step statistics and joining a Challenge), average time per session, number of pages viewed, average number of pages viewed per session, average number of step log entries, and average daily step count on days when steps were logged. Participating in individual Challenges and team Tournaments and receiving or sending friend requests were all dichotomized as *yes* or *no* (yes=participated in ≥1 Challenge or Tournament; yes=received or sent ≥1 friend request). Any average daily step count of more than 20,000 steps was truncated to 20,000 steps (n=1147) [18]. Nonusage attrition was classified as having no data entries for ≥14 consecutive days. The time to nonusage attrition was calculated using data exported from the website [23].

### Statistical Analysis

Results were expressed as differences in least square group means (odds ratios [ORs]) or hazard ratios (HRs) with 95% CIs. α was set at .05 for all analyses, which were conducted using Stata V15.1 (StataCorp LLC). in August 2019 [24]. Summary statistics were used to describe user characteristics. Group comparisons regarding age were assessed using 1-way analysis of variance with Bonferroni correction. Group comparisons regarding gender and how users found the program were assessed using chi-square tests.

Generalized linear models were used to examine differences between user groups in website sessions, minutes per session, page views, pages per session, daily step count, and total step log entries. Negative binomial family and identity link functions were used for all analyses except step count, which used a Poisson family and identity link model. The specification of the family and link functions was informed by residual diagnostics. All models were adjusted for participation in Challenges and Tournaments, as these features have been shown to be associated with usage in previous studies of 10,000 Steps users [18,25].

Associations between user groups in participation in Challenges and Tournaments and receiving and sending friend requests were assessed using binary logistic regression. These models were adjusted for Challenges and/or Tournaments depending on the outcome examined (eg, when Challenges were the outcome, model adjusted for participation in Tournament; when Tournaments were the outcome, model adjusted for participation in Challenges).

Usage and engagement with the 10,000 Steps program have been shown to be affected by participation in Challenges and Tournaments [18,25]. Consequently, 2 Cox proportional hazards regressions were conducted to assess between-group differences in time to nonusage attrition, first using an unadjusted model and then using a model adjusting for participation in Challenges and Tournaments. User group time to nonusage attrition was plotted using Kaplan-Meier survival estimates and adjusted survival curves. No obvious violations were observed upon examination of Schoenfeld residuals.

## Results

### Overview

Of the 30,040 participants’, whose mean age was 40 years, 20,992 (69.88%) were women and 7898 never logged steps (Table 1).

**Table 1:** Descriptive summary of new users—by user group—of the 10,000 Steps program registered between January 1 and November 30, 2018 (N=30,040).

Characteristics		Website Only (n=14,617)	App Only (n=2100)	Fitbit Only (n=1705)	Web and App (n=2057)	Fitbit Combination (n=1663)	Nonlogger^a^ (n=7898)
Age (years)^b,c^, mean (SD)		40.45 (12.5)	37.7 (11.8)	39.9 (11.3)	38.9 (11.6)	40.5 (12.0)	41.0 (14.0)
**Gender^d^, n (%)**							
	Female	9837 (67.3)	1363 (65)	1279 (75)	1391 (67.6)	1285 (77.3)	5837 (73.9)
	Male	4620 (31.6)	725 (34.5)	409 (24)	653 (31.8)	367 (22.1)	2015 (25.5)
	Other	160 (1.1)	12 (0.6)	17 (1)	13 (0.6)	11 (0.7)	46 (0.6)
Ever had a website session, n (%)		14,355 (98.2)	1938 (92.3)	1696 (99.5)	2028 (99.5)	1645 (98.6)	4046 (51.2)
Number of website sessions, mean (SD)		18.1 (22.8)	10.9 (20.4)	33.5 (46.3)	21.7 (27.4)	31.5 (40.5)	1.8 (2.0)
Session duration (min/session), mean (SD)		5.9 (4.9)	5.2 (6.0)	5.4 (4.5)	5.8 (4.6)	6.0 (4.4)	7.2 (8.7)
Number of page views, mean (SD)		130.7 (144.3)	50.6 (73.3)	199.5 (233.0)	133.6 (160.6)	213.0 (240.0)	16.3 (21.5)
Number of pages viewed per session, mean (SD)		9.1 (5.5)	6.7 (4.3)	7.9 (5.5)	7.9 (4.6)	8.6 (4.7)	9.9 (8.1)
Number of step log entries, mean (SD)		31.45 (24.7)	32.7 (25.1)	40.6 (36.4)	41.0 (33.6)	54.9 (49.4)	0 (0.0)
Mean daily steps^e^, mean (SD)		10,957 (4172)	10,773 (4038)	10,553 (3699)	11,307 (3887)	11,145 (3516)	0 (0.0)
Participated in at least 1 Challenge, n (%)		353 (2.4)	33 (1.6)	125 (7.3)	110 (5.3)	157 (9.4)	1 (<0.1)
Participated in at least 1 Tournament, n (%)		10,740 (73.5)	1635 (77.9)	1323 (77.6)	1691 (82.2)	1370 (82.4)	12 (0.15)
Sent or received in at least one friend request, n (%)		1956 (13.4)	247 (11.8)	363 (21.3)	374 (18.2)	334 (20.1)	1 (0.01)

^a^Some users engaged with program content but never logged steps.

^b^For the *Age* row, n=29,961 because of missing values.

^c^Indicates a significant difference (*F*_5_=26.84; *P*<.001) between usage groups with respect to age.

^d^Indicates a significant difference (*X^2^*_10_=216.7; *P*<.001) between usage groups with respect to gender.

^e^The mean daily steps include steps allocated as a result of additionally recorded moderate or vigorous activity and/or distance.

### User Characteristics

Table 1 shows a descriptive summary of user groups related to age and gender. App Only users were significantly younger than all other groups (Website Only: *P*<.001; Fitbit Only: *P*<.001; Fitbit Combination: *P*<.001; Nonloggers: *P*<.001; Web and App users: *P*=.05). Web and App users were significantly younger than Website Only users (*P*<.001), Fitbit Combination users (*P*=.001), and Nonloggers (*P*<.001). Nonloggers were significantly older than all other groups except Fitbit Combination users (Website Only: *P*=.01; App Only: *P*<.001; Fitbit Only: *P*=.01; Web and App: *P*<.001). Although statistically significant differences in age were found, they are unlikely to be meaningful because the difference in years was small. Gender was significantly different between user groups with the highest proportions of women in the 2 groups that used Fitbits (Fitbit Only: 1279/1705, 75.01%; Fitbit Combination: 1285/1663, 77.27%).

### Between-Group Differences in Engagement With the 10,000 Steps Program

The between-group differences in program engagement metrics from January 1 to December 31, 2018, are shown in Table 2. On average, the App Only group had significantly fewer website sessions (mean difference [MD] −6.9; 95% CI −7.6 to −6.2), whereas the Fitbit Only (MD 10.6; 95% CI 8.8-12.3), Web and App (MD 1.5; 95% CI 0.4- 2.6), and Fitbit Combination (MD 8.0; 95% CI 6.2- 9.7) groups had significantly more website sessions, relative to the Website Only group. The App Only and Fitbit Only groups spent significantly fewer minutes (MD −0.7; 95% CI −0.9 to −0.4; MD −0.5; 95% CI −0.7 to −0.2, respectively), whereas the Fitbit Combination group spent significantly more minutes (MD 0.2; 95% CI 0.0-0.5) on the website for each session, relative to the Website Only group. These differences, although statistically significant, were small in magnitude (<1 min per session).

**Table 2:** Marginalized means and between–user group differences for website usage and mean daily step count and logging between January 1 and December 31, 2018.

Website usage parameters, step counts, and step log entries^a,b^ by group		Value, mean (95% CI)	Between-group coefficient (95% CI)	*P* value^c^
**Total number of sessions^d,e^**				
	Website Only	18.8 (18.4 to 19.1)	Reference category	N/A^f^
	App Only	11.9 (11.2 to 12.6)	−6.9 (−7.6 to −6.2)	<.001
	Fitbit Only	29.4 (27.7 to 31.1)	10.6 (8.8 to 12.3)	<.001
	Web and App	20.2 (19.2 to 21.3)	1.5 (0.4 to 2.6)	.01
	Fitbit Combination	26.8 (25.1 to 28.5)	8.0 (6.2 to 9.7)	<.001
**Minutes per session^d,e^**				
	Website Only	5.9 (5.8 to 6.0)	Reference category	N/A
	App Only	5.2 (5.0 to 5.5)	−0.7 (−0.9 to −0.4)	<.001
	Fitbit Only	5.4 (5.2 to 5.6)	−0.5 (−0.7 to −0.2)	<.001
	Web and App	5.9 (5.7 to 6.1)	0.1 (−0.1 to 0.3)	.54
	Fitbit Combination	6.1 (5.9 to 6.3)	0.2 (0.0 to 0.5)	.03
**Total number of page views^e^**				
	Website Only	132.3 (130.2 to 134.3)	Reference category	N/A
	App Only	62.8 (59.7 to 65.8)	−69.5 (−72.9 to −66.1)	<.001
	Fitbit Only	179.9 (171.3 to 188.5)	47.6 (38.8 to 56.5)	<.001
	Web and App	123.6 (118.4 to 128.8)	−8.6 (-14.2 to −3.1)	.002
	Fitbit Combination	183.0 (175.1 to 191.0)	50.8 (42.5 to 59.0)	<.001
**Number of pages viewed per session^d,e^**				
	Website Only	9.0 (8.9 to 9.1)	Reference category	N/A
	App Only	6.8 (6.6 to 7.0)	−2.2 (−2.4 to −2.0)	<.001
	Fitbit Only	7.9 (7.6 to 8.1)	−1.2 (−1.4 to −0.9)	<.001
	Web and App	8.0 (7.8 to 8.2)	−1.0 (−1.2 to −0.8)	<.001
	Fitbit Combination	8.9 (8.7 to 9.1)	−0.1 (−0.4 to 0.1)	.24
**Mean daily step count ^g^**				
	Website Only	10,987 (10,920 to 11,055)	Reference category	N/A
	App Only	10,785 (10,612 to 10,958)	−201.9 (−387.7 to −116.0)	.03
	Fitbit Only	10,494 (10,320 to 10,669)	−492.9 (−679.9 to −305.8)	<.001
	Web and App	11,245 (11,077 to 11,413)	258.0 (76.9 to 439.2)	.005
	Fitbit Combination	10,996 (10,826 to 11,166)	9.1 (−174.1 to 192.3)	.92
**Number of step log entries^e^**				
	Website Only	32.3 (31.9 to 32.7)	Reference category	N/A
	App Only	33.5 (32.3 to 34.6)	1.2 (−0.1 to 2.4)	.07
	Fitbit Only	37.3 (35.8 to 38.8)	5.0 (3.4 to 6.6)	<.001
	Web and App	39.5 (38.2 to 40.8)	7.2 (5.9 to 8.6)	<.001
	Fitbit Combination	47.9 (46.1 to 49.7)	15.6 (13.7 to 17.5)	<.001

^a^Between user groups; Website Only group: n=14,617; App Only group: n=2100; Fitbit Only group: n=1705; Web and App group: n=2057; Fitbit and web and/or app group: n=1663.

^b^Website usage: website usage engagement may not have occurred on consecutive days.

^c^α=.05.

^d^Session: using the website to interact with content. For example, viewing step statistics and joining a Challenge.

^e^Model based on generalized linear regression using negative binomial family and identity link, which was adjusted for Challenge and Tournament counts.

^f^N/A: not applicable.

^g^Model based on generalized linear regression using Poisson family and identity link, which was adjusted for Challenge and Tournament counts.

Significantly fewer website pages were viewed by the App Only (MD −69.5; 95% CI −72.9 to −66.1) and Web and App (MD −8.6; 95% CI −14.2 to −3.1) groups relative to the Website Only group, whereas significantly more pages were viewed by the Fitbit Only (MD 47.6; 95% CI 38.8 to 56.5) and Fitbit Combination (MD 50.8; 95% CI 42.5 to 59.0) groups. All groups, except the Fitbit Combination group, viewed significantly fewer website pages per session than the Website Only group.

The mean daily step count was significantly lower for the App Only (MD −201.9; 95% CI −387.7 to −116.0) and Fitbit Only (MD −492.9; 95% CI −679.9 to −305.8) groups relative to the Website Only group, whereas the mean daily step count of the Web and App group (MD 258.0; 95% CI 76.9- 439.2) was significantly higher. The App Only and Website Only groups did not significantly differ from each other in the number of step log entries, whereas the Fitbit Only, Web and App, and Fitbit Combination groups all logged steps a significantly greater number of times (MD 5.0, 95% CI 3.4-6.6; MD 7.2, 95% CI 5.9-8.6; and MD 15.6, 95% CI 13.7-17.5, respectively) than the Website Only group, with the Fitbit Combination group logging steps the highest number of times.

The App Only group was less likely (Challenges: OR 0.65, 95% CI 0.46-0.94; friend requests: OR 0.86, 95% CI 0.75-0.99) and all other groups were significantly more likely (Challenges: OR 2.38-4.48; *P*<.001; friend requests: OR 1.37-1.67; *P*<.001) than the Website Only group to participate in Challenges as well as send and/or receive friend requests. All groups were more likely than the Website Only group to participate in Tournaments (OR 1.26-1.79; *P*<.001; Table 3).

**Table 3:** Associations between participating in Challenges and Tournaments and receiving and sending friend requests from January 1 to December 31, 2018 among the 10,000 Step user groups.

User groups^a^		OR^b^ (95% CI)	*P* value
**Participating in individual Challenges^c^ (reference category: no)**			
	Website Only	Reference group	N/A^d^
	App Only	0.65 (0.46-0.94)	.02
	Fitbit Only	3.29 (2.66-4.06)	<.001
	Web and App	2.38 (1.91-2.97)	<.001
	Fitbit Combination	4.48 (3.68-5.46)	<.001
**Participating in team Tournaments^e^ (reference category: no)**			
	Website Only	Reference group	N/A
	App Only	1.26 (1.13-1.41)	<.001
	Fitbit Only	1.28 (1.14-1.45)	<.001
	Web and App	1.70 (1.51-1.91)	<.001
	Fitbit Combination	1.79 (1.57-2.05)	<.001
**Receiving and/or sending friend requests^f^ (reference category: no)**			
	Website Only	Reference group	N/A
	App Only	0.86 (0.75-0.99)	.04
	Fitbit Only	1.67 (1.47-1.89)	<.001
	Web and App	1.37 (1.21-1.54)	<.001
	Fitbit Combination	1.44 (1.27-1.65)	<.001

^a^Website Only group: n=14,617; App Only group: n=2100; Fitbit Only group: n=1705; Web and App group: n=2057; Fitbit Combination group: n=1663.

^b^OR: odds ratio.

^c^Adjusted for Tournaments.

^d^N/A: not applicable.

^e^Adjusted for Challenges.

^f^Adjusted for Tournaments and Challenges.

### Nonusage Attrition

**Figure 2:**
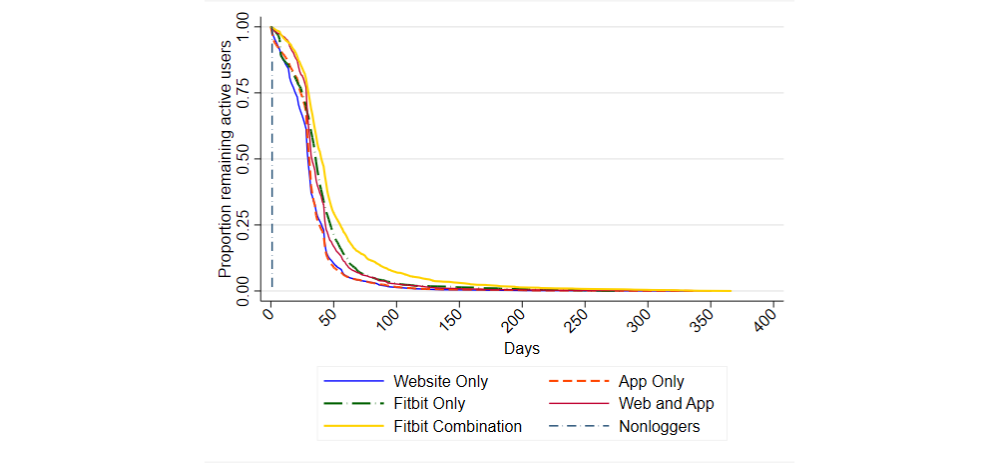
Kaplan-Meier estimates of the survival distribution for time to nonusage attrition by group.

**Figure 3:**
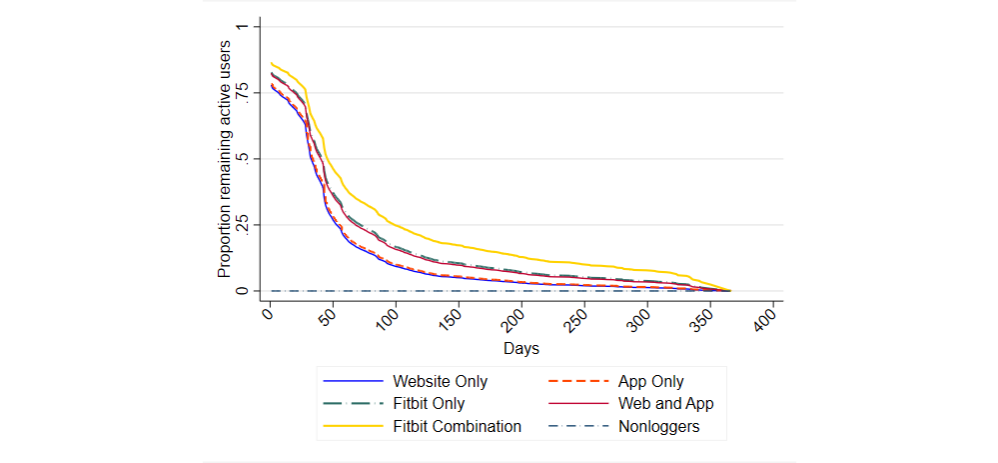
Cox proportional hazards regression curve of the survival distribution for time to nonusage attrition by group based on the model adjusted for Challenges and Tournaments; reference category: did not participate in Challenges or Tournaments.

Of the 22,142 new users who logged steps at least once, 21 (0.09%) did not succumb to nonusage attrition between the time of registration and December 31, 2018. The mean time to nonusage attrition was 35 (SD 26) days (Website Only: mean 32 days, SD 22 days; App Only: mean 33 days, SD 23 days; Fitbit Only: mean 40 days, SD 29 days; Web and App: mean 39 days, SD 27 days; Fitbit Combination: mean 50 days, SD 40 days). Among those who logged steps at least once, the estimated median time to nonusage (ie, the time after which 50% of users cease logging steps) was 31 days, with the Fitbit Combination group taking the longest time to reach this point (41 days; Table 4). All groups, except the App Only group, had a significant difference in time to nonusage attrition relative to the Website Only group (HR: range 0.55-0.75), and this association remained, although slightly attenuated, after adjusting for participation in Challenges and Tournaments (HR: range 0.59-0.78; Table 5; Figure 2, Figure 3). The Fitbit Combination group had a >40% lower likelihood of succumbing to nonusage attrition than the Website Only group.

**Table 4:** Cox proportional hazard risks for nonusage attrition of 10,000 Steps program, by user group and by participation in Challenges and Tournaments (N=30,040).

Group/participation		Unadjusted^a^ HR^b^ (95% CI)	*P* value	Adjusted^c^ HR (95% CI)	*P* value
**User group**					
	Website Only	Reference group	N/A^d^	N/A	N/A
	App Only	0.97 (0.93-1.01)	.17	0.97 (0.93-1.02)	.22
	Fitbit Only	0.71 (0.68-0.75)	<.001	0.75 (0.72-0.79)	<.001
	Web and App	0.75 (0.72-0.79)	<.001	0.78 (0.74-0.81)	<.001
	Fitbit Combination	0.55 (0.52-0.57)	<.001	0.59 (0.56-0.62)	<.001
**Level of participation in Challenges and Tournaments**					
	Did not participate in Challenge	Reference group	N/A	N/A	N/A
	Participated in Challenge	0.48 (0.45-0.52)	<.001	N/A	N/A
	Did not participate in Tournament	Reference group	N/A	N/A	N/A
	Participated in Tournament	0.75 (0.73-0.78)	<.001	N/A	N/A

^a^Unadjusted model.

^b^HR: hazard ratio.

^c^Model adjusted for participation in Challenges and Tournaments.

^d^N/A: not applicable.

**Table 5:** Survival time by group of users of the 10,000 Steps program (N=30,040).

Percentage of user groups still using the 10,000 Steps platform^a^		Duration (days)
**75%**		
	Website Only	20
	App Only	24
	Fitbit Only	25
	Web and App	29
	Fitbit Combination	30
	Nonloggers	0
	All logging groups combined	22
	All groups combined	0
**50%**		
	Website Only	30
	App Only	31
	Fitbit Only	36
	Web and App	33
	Fitbit Combination	41
	Nonloggers	0
	All logging groups combined	31
	All groups combined	27
**25%**		
	Website Only	41
	App Only	39
	Fitbit Only	48
	Web and App	44
	Fitbit Combination	56
	Nonloggers	0
	All logging groups combined	43
	All groups combined	37

^a^Participants may not have engaged with the program on all days consecutively.

## Discussion

### Principal Findings

This study examined whether different methods of logging steps in the 10,000 Steps physical activity program are associated with engagement with the program. The results showed that those who used Fitbits (Fitbit Only and Fitbit Combination) were the most engaged with the program. There was also some evidence that those who used the combination of Web and App were also well engaged with the program, but not to the same extent as Fitbit users.

The Fitbit Combination group had an engagement profile that included a high number of website sessions, page views, and step log entries as well as a higher likelihood of participating in both individual Challenges and team Tournaments. This group also had the lowest risk of succumbing to nonusage attrition, which corresponded with the longest time to succumb to nonusage attrition. The Fitbit Only group also had relatively good engagement characteristics and logged steps for the second longest time. These results suggest that the use of a Fitbit may enhance engagement with a real-world physical activity program. This also indicates that using a Fitbit in combination with other step-logging methods may further enhance engagement.

These findings were not expected because Fitbit users had the option to view their step counts either on the Fitbit itself or on the Fitbit website or app. Subsequently, they could have neglected to log in to the 10,000 Steps website to synchronize their Fitbit steps and interact with the other program content on the website. A number of factors may partially explain the superior engagement of the groups using Fitbits. The financial investment involved in purchasing a Fitbit may indicate that these device-owning individuals were more motivated to increase their activity levels compared with the other groups. Previous research has suggested that monetary investment may be associated with behavior change either because of the presence of motivation at the time of purchase or through financial investment stimulating motivation [26]. Advanced activity tracking devices have previously been shown to reduce the burden of self-monitoring activity compared with traditional self-monitoring methods such as pedometers [27]. The increased convenience may lead to improved engagement with other program features (eg, reading website content). In addition, wearing a Fitbit may serve as an activity-related prompt or cue. Another factor may include the nature of the Fitbits themselves. Users of activity tracking devices have cited a number of features that apply to Fitbits as being important characteristics, such as being wrist-worn; being accurate [28]; having the ability to synchronize with other devices; and having the ability to track additional items such as heart rate, distance traveled, and sleep [29,30]. Given that the level of engagement with activity trackers is associated with high user satisfaction [29] these factors may act as motivators for users to maintain program engagement and be active. Other features of Fitbits that may improve engagement within these groups include the associated Fitbit app, which may have increased exposure to additional motivational messaging and the incorporation of behavior change techniques such as goal setting and feedback [8]. Engagement may be active (ie, logging data or completing quizzes) or passive (viewing the intervention without interacting) [31], and it is unclear how Fitbit use may influence this as the data are automatically synchronized to the 10,000 Steps platform. This aspect of engagement with a Fitbit may be passive relative to the manual entry of data. Furthermore, differences in broader indicators of use, such as session duration and page views, were not consistent between the groups that included Fitbit. Consequently, it may be interesting for future studies to examine the mode of self-monitoring in the context of a broader set of engagement indicators.

The Fitbit Combination group was less likely to succumb to nonusage attrition and took longer to succumb to nonusage attrition. This might reflect research showing that behavior change and the sustained use of activity trackers are enhanced when trackers (such as Fitbit) that provide feedback are used in conjunction with other interventions (such as the 10,000 Steps program), which delineate target behaviors and provide a plan of action [32]. In addition, research exploring the relationship between the use of a Fitbit and changes in physical activity suggested that being accountable to someone else had a greater influence on increasing physical activity than simply self-monitoring the data on a Fitbit [27]. A sense of accountability might have been created through registration with the 10,000 Steps program and by the greater participation in individual Challenges and team Tournaments among the Fitbit Combination group. Furthermore, team Tournaments involving social interaction are associated with improved adherence to physical activity interventions [33].

A higher proportion of women were found in the 2 groups that used Fitbits. This is contrary to previous research showing that men were more likely to use advanced activity trackers [29]. Previous research also indicated that women were more likely to use any type of activity tracker [29]; therefore, perhaps the fast-growing adoption of advanced activity trackers is now greater in women. It has been shown that the sustained use of activity trackers is longer among women than in men [32], and this factor might have contributed to the longer time to nonusage among the Fitbit Combination group, which had the highest proportion of women.

The App Only group was significantly younger, with a higher proportion of men than the other groups. This group demonstrated the lowest overall interaction with program content and was among the groups with the lowest mean daily step count and the earliest time to nonusage attrition. These findings suggest that additional strategies may be required to better engage younger male users of the program. Interestingly, 2 previous studies of the 10,000 Steps programs, which also examined app use, found contrasting outcomes [18,34]. The first study found the App Only group to be the youngest but mostly female, with engagement and time to nonusage attrition being better than those in the Website Only group [18]. The second study, which was undertaken when the app was first introduced, examined the app and web users of the 10,000 Steps program and found that app users logged more steps more often than Website Only users [34]. These discrepancies in findings could potentially be explained by those users who tend to be the *early adopters* of innovations [35]: those who were *early adopters* of the app when it was first introduced may have similar characteristics to those who are now the *early adopters* of the Fitbit functionality.

The groups that used a combination of step-logging methods had the best program engagement profile. This is highlighted by the greater number of step log entries. The higher the mean daily step count, the greater the likelihood of participating in individual Challenges and team Tournaments, and the longer the time until nonusage attrition by the Fitbit Combination group compared with the Fitbit Only group and by the Web and App group compared with both the App Only and Website Only groups. This consolidates the findings from a previous study of engagement with the 10,000 Steps program that found the Web and App group to have a better engagement profile than the Website Only and App Only groups [18]. It is difficult to determine whether the act of using multiple methods to log steps maintains a longer interest in the program or whether it is a personality type or level of motivation to be active, which leads them to both use several logging methods and stay engaged.

Nonusage attrition is a common problem among internet-delivered health interventions [36]. The mean time to nonusage attrition in this sample was similar to previous studies of the 10,000 Steps program, and both studies found that groups that logged steps using multiple methods (eg, using a combination of Web and App to log steps) took longer to cease logging steps [18]. The time to nonusage attrition in this study also compared favorably with several other mobile health physical activity studies conducted under both real-world settings (35 days in this study vs 11 days in another study [37]) and tightly controlled conditions (35 days in this study vs 32 [38] and 46 days [39] in other studies).

The mean daily step counts of all the groups was greater than 10,000 Steps, which is above the average of 7400 for Australian adults [40]. Although this is a promising indicator that this real-world physical activity intervention is effective, the magnitude of differences between the groups was <500 per day. Consequently, the broader public health implications of these differences are unclear and may be more relevant and meaningful for less active people, given that they benefit most from small increases in activity [41]. Alternatively, it may reflect that more active individuals are attracted to the program. The mean daily step counts were also the highest for the groups that used more than one method to log steps. However, the Fitbit Only group took the fewest steps, including fewer steps than the Website Only and App Only groups, despite the groups’ other engagement parameters being generally better. This might also be indicative of the different impacts on behavior stemming from active versus passive self-monitoring [27]. Those required to manually enter their daily step counts may be more likely to consciously scrutinize their step counts during the process and consequently make a greater effort to take more steps. Meanwhile, the passive nature of the automatic synchronization of step counts by the Fitbit, when not linked to other interactions with program content, may lead to a loss of attention to step counts and subsequently to less stimulus to change behavior. A previous study of Fitbit users found that there were better improvements in physical activity among users who interacted with both the app and the Fitbit device than those who just checked their device [27]. Therefore, it is possible that the groups in this study who used a greater number of methods to monitor their steps might be undertaking active rather than passive self-monitoring (ie, paying attention to the feedback or graphs, etc), which led to better engagement by these groups.

### Strengths and Limitations

Among the strengths of this study is the real-world delivery of a web-based physical activity intervention, not in a controlled setting. In addition, the sample was large and examined over a long time frame. The findings of this type of study are likely to provide more accurate information regarding how interventions work when they are delivered in ecologically valid settings [6]. Other strengths include the examination of various combinations of user step-logging methods that allow for a nuanced understanding of user engagement patterns. There are several limitations to consider. The results of this study must be interpreted in the context of the vast majority of users belonging to the Website Only group, and the outcomes may have been different if the groups were more evenly balanced. In addition, the only measure of physical activity was step count, which does not necessarily capture the overall physical activity. Furthermore, the Fitbit groups provided objective step data that were automatically synced with the website, whereas the others provided manually entered pedometer measured step counts that are prone to bias (ie, people may report more than what the pedometer actually measured). This might have created a disparity in the accuracy of step counts between the groups. Only 25% (5536/22,142) of those who actively logged steps were still engaged after 43 days; therefore, it is unknown whether the impressive >10,000 daily mean step counts of all groups was ongoing. It is noteworthy that the engagement metrics used in this study did not include broader indicators of different types of engagement (reflective, altruistic, and gamified), which may be important to behavior change [31].

### Conclusions

This study found that multiple methods of logging steps were associated with better program engagement. The use of a Fitbit appears to enhance engagement with a real-world physical activity program, particularly when used in conjunction with other platforms (ie, a combination of Fitbit and website and/or app). Therefore, integrating tracking devices that synchronize data automatically into real-world physical activity interventions is one strategy to improve engagement.

## Abbreviations

HR: hazard ratio
MD: mean difference
OR: odds ratio
